# Development of Early-Life Gastrointestinal Microbiota in the Presence of Antibiotics Alters the Severity of Acute DSS-Induced Colitis in Mice

**DOI:** 10.1128/spectrum.02692-21

**Published:** 2022-04-19

**Authors:** Xiaojun Li, Yu Ren, Jie Zhang, Chunhui Ouyang, Chunlian Wang, Fanggen Lu, Yani Yin

**Affiliations:** a Department of Gastroenterology, The Second Xiangya Hospital of Central South University, and Research Center of Digestive Disease, Central South University, Changsha, Hunan, People’s Republic of China; b Department of Gastroenterology, Xiangya Hospital of Central South University, Changsha, Hunan, People’s Republic of China; c National Clinical Research Center for Geriatric Disorders, Xiangya Hospital, Changsha, Hunan, People’s Republic of China; d Hunan International Scientific and Technological Cooperation Base of Artificial Intelligence Computer Aided Diagnosis and Treatment for Digestive Disease, Changsha, Hunan, People’s Republic of China; USDA-ARS, ACNC

**Keywords:** 16S rRNA, antibiotic use, gut microbiota, lactation, mouse models, prenatal

## Abstract

Early-life gastrointestinal microbiota development is crucial for physiological development and immunological homeostasis. In the current study, perinatal microbiota and the development of gastrointestinal microbiota in different early-life periods (perinatal, lactation, and postweaning nutrition periods) were explored by using an antibiotic-interfered mouse model and a dextran sulfate sodium-induced colitis mouse model. Gut microbiota samples were collected from mother mice and litters. The results of 16S rRNA gene sequences suggested that microbiota in the gastrointestinal system were present in prenatal fetal mice, and microbiota structures in different parts of the gastrointestinal system of the fetal mice were similar to those in the corresponding gut parts of maternal mice. Microbiota in mucus samples from different regions exhibited higher diversity at birth than at other periods and varied substantially over time with diet change. Moreover, antibiotic treatment in early life affected the composition and diversity of gastrointestinal microbiota in adult mice and enhanced susceptibility to experimental colitis in mice, particularly in the lactation period. This approach of exploring gut microbiota evolution is hoped to provide an enhanced view of how resident microbiota develop in early life, which in turn might facilitate understanding of gut microbiota and related diseases.

**IMPORTANCE** This study investigated resident microbiota in the whole gastrointestinal (GI) tract to explore gut microbiota development in early life and found that early-life antibiotic exposure exacerbated alterations in gut microbiota and murine dextran sulfate sodium (DSS)-induced colitis. Furthermore, the presence of bacteria in the GI tract of mice before birth and the importance of the lactation period in GI microbiota development were confirmed.

## INTRODUCTION

Gut microbiota dysbiosis is commonly observed in inflammatory bowel disease (IBD) and appears to play a pathological role in the initiation and progression of this disease (1–3). Most patients with IBD present with different degrees of dysbiosis during the active phase of disease, and microbiome-based therapeutics, such as prebiotics, probiotics, and fecal microbiota transplantation, have been proposed as effective treatments for patients with IBD (4–6). Consequently, gastrointestinal (GI) microbiota is regarded as an important factor in development of intestinal diseases. In both mice and humans, the GI microbiota development occurs predominantly in early-life periods, which are crucial for the microbiota maturation, and remains comparatively stable from that point onwards (7, 8). The mode of delivery, breastfeeding, the environment, and the use of antibiotics in early life can affect GI microbiota development, and these factors occur mostly in perinatal and neonatal periods (9). Most of these factors have been recognized as potential triggers for IBD, nevertheless, related researches have focused mainly on the postnatal growth period (10–12). The early-life period includes the pregnancy period and the first 2 years of life, which can be classified into three stages: perinatal, lactation, and postweaning nutrition period. Despite the strong association between the impacts of early-life intervention factors on microbial maturation and future risk of IBD, the main roles of each stage of the early-life period in the process of intestinal microbiota development have yet to be elucidated.

In past decades, the prenatal environment was recognized as sterile in healthy individuals, and a mother’s microbiota (vaginal and perineal) were considered the initial seeding bacteria that shaped newborn gut microbiota colonization (13). However, the rapid development of sequencing technology has revealed the presence of bacterial microbiota in placental specimens from healthy pregnant women, challenging the dogma of a sterile womb (14). Multiple broad-range studies have provided evidence for the presence of microbiota at prenatal sites. However, some researchers pointed out a contradiction between *in utero* colonization and the ability to generate germfree mammals. Instead, they identified contamination as the major factor in the prenatal microbiota (15). Therefore, dissecting the development stage of the first seeding bacteria in infants and the association in the mother-newborn interphase is crucial.

The mode of delivery, breastfeeding, diet, and the use of antibiotics in early life were considered to influence GI microbiota development, and current studies have focused mostly on the postnatal period (16–20). After birth, gut microbiota formation was gradually influenced by the factors mentioned above and finally matured to a distinct adult-type microbiota. Due to the different delivery modes at birth, microbiota of vaginally delivered newborns resembled maternal vaginal microbiota, which is dominated by species of the genera *Lactobacillus* and *Prevotella*, while neonates delivered by caesarean section appear to contain microbiota similar to that of the maternal skin, including *Staphylococcus*, *Corynebacterium*, and *Propionibacterium* (17). Breastfeeding, a key factor that supports adequate microbial colonization, universally features *Staphylococcus*, *Streptococcus*, and *Propionibacterium* and also contains a wide variety of bioactive compounds to satisfy the needs of a growing infant in the lactation period (19, 21). After breastfeeding, dietary structure becomes the main factor in the postweaning nutrition period. Mounting evidence indicates that the Western diet, containing high protein, sugar, or fat, affects the diversity and populations of GI microbial species profoundly (22). Antibiotic intake, as one of the most common treatments in early life, is a postnatal factor that can influence the establishment of infant microbiota. Although antibiotics are usually administered short-term, considering the critical time of newborn gut bacteria acquisition, they can be used as an intervention throughout early life. There are abundant lines of evidence suggesting that antibiotic exposure during the early-life period may affect both the gestational process and the composition of the microbiota, which may further influence host health status and play a role in IBD (12, 23–25). Since numerous factors affect the gut microbiota development, the most susceptible stage in early life to antibiotics still needs to be clarified.

The current study aimed to understand the complex development of gut microbiota in different stages of early life. Neonatal GI microbiota of mice were observed, and mice models with antibiotic interference at different stages of early life were established. The mice were subsequently treated with dextran sulfate sodium (DSS) to induce acute colitis later in life. Susceptibility to colitis and compositional alterations of fecal and mucosa-associated microbiota were assessed. This approach of exploring GI microbiota evolution allowed investigation of the period and mode of susceptibility to colitis and could enhance understanding of GI microbiota development and related diseases.

## RESULTS

### Maternal microbiota and fetal gastrointestinal microbiota in mice.

The 16S rRNA gene sequencing results showed the presence of bacteria in fetal mice before birth. A group of pregnant mice were sacrificed on the expected date of confinement before giving birth, and gastric mucus, small intestine mucus, and large intestine mucus from fetal (*n* = 5) and pregnant mice (*n* = 5), plus stools and placenta from pregnant mice, were collected. The microbiota structures in these parts of fetal mice were similar to those in corresponding parts of maternal mice.

**FIG 1:**
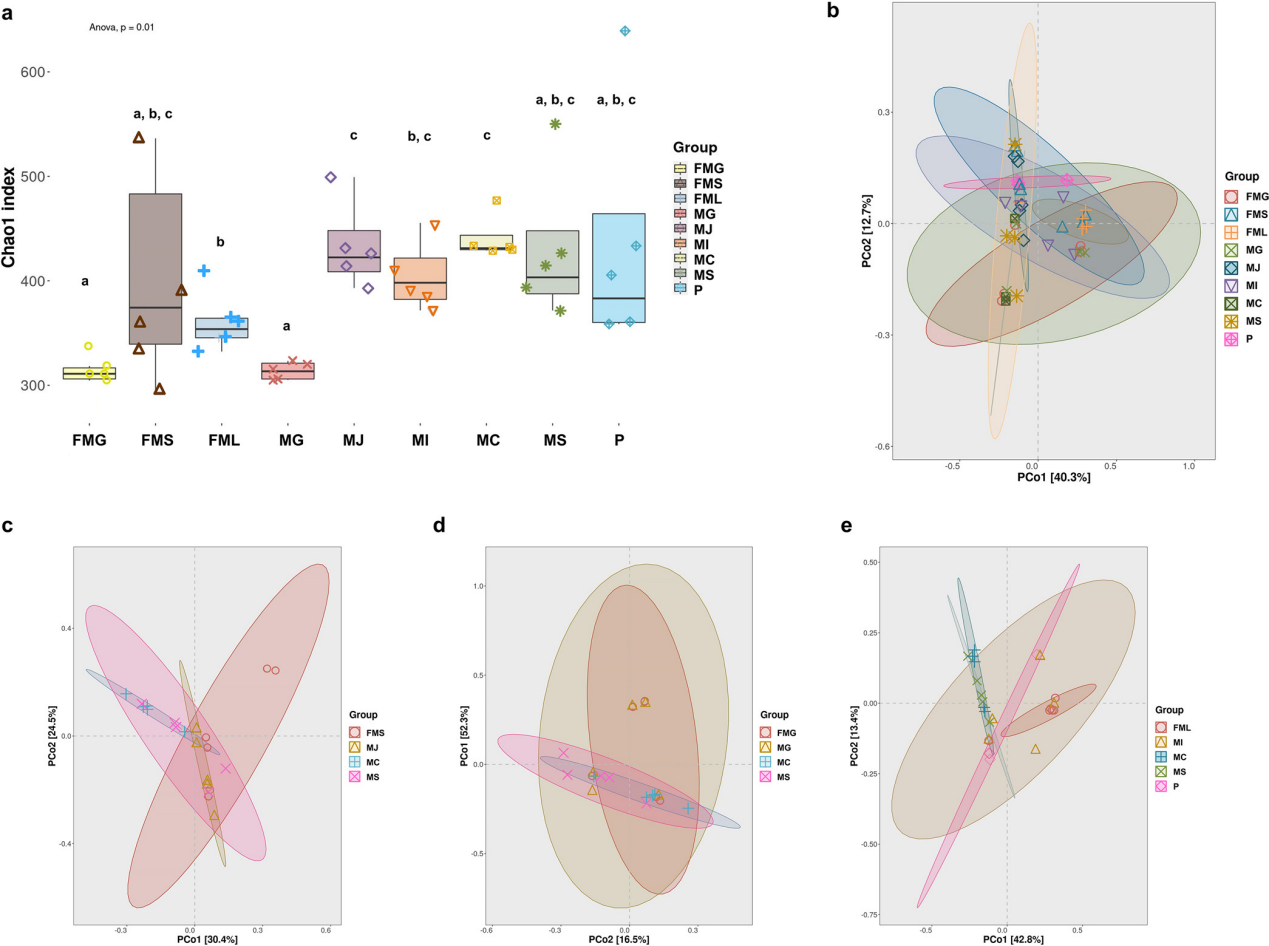
Alpha diversity and beta diversity of maternal microbiota and fetal gastrointestinal microbiota in mice. (a) Alpha diversity of maternal and fetal mice samples was calculated by Chao1 index. Beta diversity was calculated among different groups as shown in panels b to e. MG, maternal gastric mucus group; MJ, maternal jejunum mucus group; MI, maternal ileum mucus group; MC, maternal cecum mucus group; MS, maternal stool group; P, placenta group; FMG, fetal mice gastric group; FMS, fetal mice small intestine group; FML, fetal mice large intestine group. Data are shown as the mean ± standard error of the mean (SEM). Groups marked with the same lowercase letter were not significantly different from one another, and those marked with different letters differed significantly (*P* < 0.05) by Kruskal–Wallis tests and one-way analysis of variance (ANOVA).

**Microbiota diversity was lower in gastric mucus than in GI parts in fetal mice.** Chao1 richness estimator, Simpson’s index, and Shannon index were measured to evaluate the richness, evenness, and alpha diversity (α-diversity) of microbiota. As shown in Fig. 1a and Fig. S1, all α-diversity indices of the fetal mice gastric group (FMG) were significantly lower than those of the fetal mice large intestine group (FML) (*P* < 0.05), while no significant differences were observed in fetal mice small intestine (FMS) compared to the other two groups. Similarly, maternal gastric mucus group (MG) showed α-diversity indices lower than those of the other maternal mice GI parts (*P* > 0.05). Microbiota structure beta diversity (β-diversity) was analyzed with unweighted UniFrac principal coordinate analysis (PCoA) (Fig. 1b to e). The gastric microbiota in maternal and fetal mice showed similarity and presented an overlap between two groups but were obviously different from other GI parts. The FMS and FML groups overlapped with maternal jejunum mucus (MJ), maternal ileum mucus (MI), and placenta (P) groups, which were clustered and separate from the maternal cecum mucus (MC) and maternal stool (MS) groups, as evidenced by the clustering of samples in plots.

**FIG 2:**
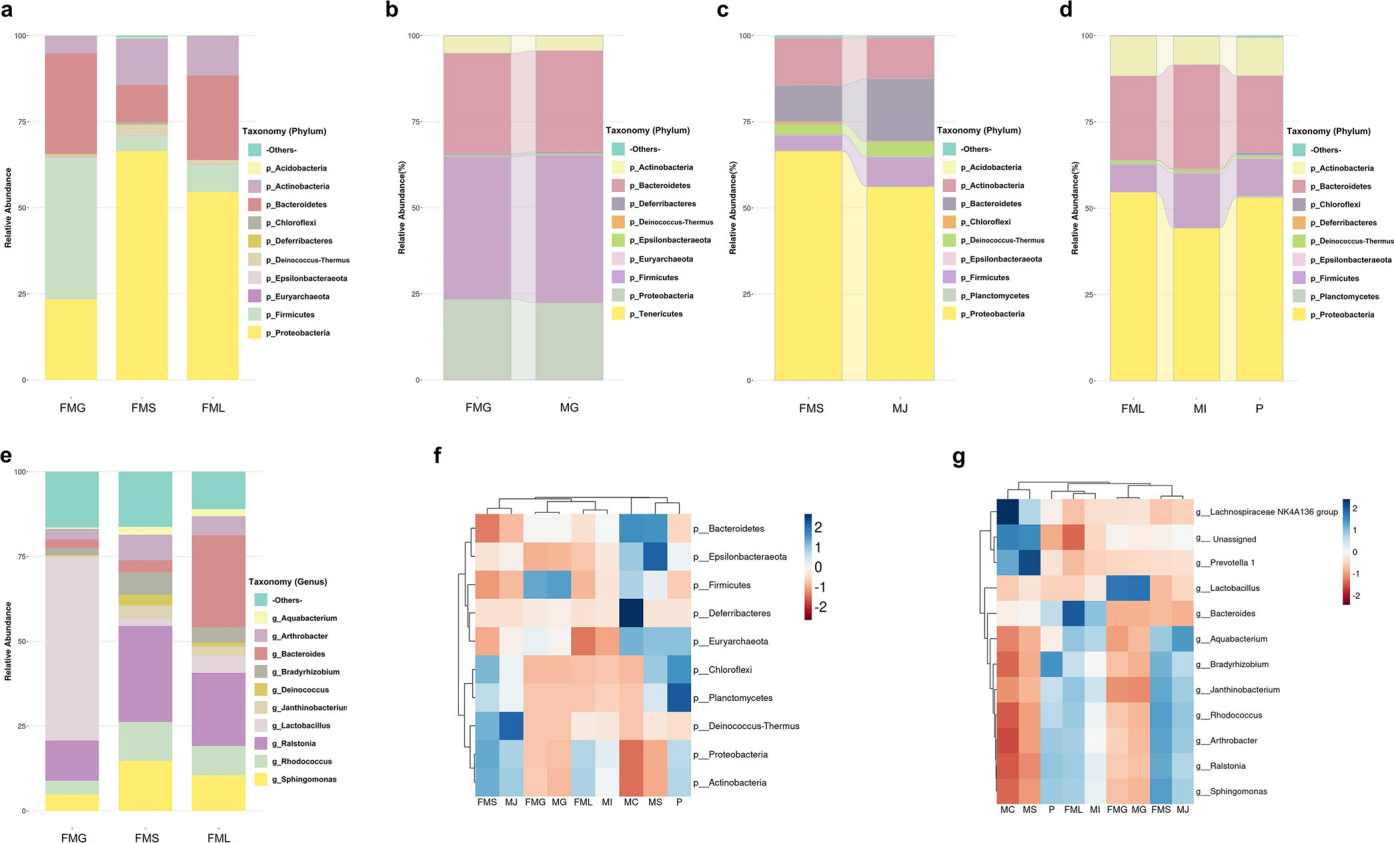
Microbiota composition of maternal and fetal gastrointestinal bacteria in mice. (a) Microbiota composition of fetal mice gastric (FMG), fetal mice small intestine (FMS), and fetal mice large intestine (FML) at the phylum level. (b) The relative abundance of bacteria in FMG and maternal gastric mucus (MG) at the phylum level. (c) The relative abundance of bacteria in FMS and maternal jejunum mucus (MJ) at the phylum level. (d) The relative abundance of bacteria in FML, maternal ileum mucus (MI), and placenta (P) at the phylum level. (e) Microbiota composition of FMG, FMS, and FML at the genus level. (f) Clustered heat maps of relevance values across maternal and fetal mice samples at phylum level and calculations by a Z-score, and lines represent the specified distance or similarity value. (g) Clustered heat maps of relevance values across maternal and fetal mice samples at genus level.

**The microbiota structures in each GI site of fetal mice were similar to corresponding parts of maternal mice.** Microbiota characteristics of the GI tract in fetal mice before birth were evaluated by the relative abundance of predominant taxa. The most abundant taxa at the phylum and genus levels are shown in Fig. 2. A total of 10 phyla were identified in fetal mice groups (Fig. 2a). The three most abundant phyla in the FMG were *Firmicutes* (41.5 ± 8.14%), *Bacteroidetes* (28.8 ± 9.13%), and *Proteobacteria* (23.5 ± 10.66%), while the three dominant phyla in FMS and FML groups were *Proteobacteria* (66.6 ± 6.35%, 54.3 ± 11.27%), *Bacteroidetes* (10.6 ± 5.81%, 24.7 ± 12.97%), and *Actinobacteria* (13.5 ± 2.58%, 11.7 ± 3.93%). The relative abundance of *Bacteroidetes* in FMG was significantly higher than that in the other parts, while the relative abundances of *Proteobacteria* and *Actinobacteria* were lower.

At the genus level (Fig. 2e), the relative abundance of the genus *Lactobacillus* was significantly higher in the FMG than in other parts (*P* < 0.05). The relative abundance gradually decreased along the lower GI tract. In contrast, the relative abundance of *Bacteroides* progressively increased along the lower GI tract. Calculations of a Z-score for each group indicated that microbiota structures were similar at the genus level between the small intestine and large intestine mucus of fetal mice.

To understand the potential bacterial transmission in the maternal-fetal interphase, maternal mice microbiota were compared with fetal mice microbiota. A total of 12 phyla were identified (Fig. 1b to d). The overall microbiota structure of the FMG was similar to that of the MG, with a predominance of *Firmicutes*, *Bacteroidetes*, and *Proteobacteria*. Coincidentally, the overall microbiota structure of the FMS and FML was similar to that of MJ and MI, respectively. Microbiota of the placenta exhibited a higher relative abundance of *Chloroflexi* and *Planctomycetes* than the other maternal mice groups (Fig. 2f). At the genus level, FMG and FMS again presented microbiota structures similar to those of the corresponding gut parts in maternal mice (Fig. 1g, Fig. S2).

### Gastrointestinal microbiota development in different periods of early life.

Meconium and mucus from the stomach, ileum, and cecum of neonatal mice were collected at 2 h within birth (0W) (*n* = 5), 2 weeks old (2W) (*n* = 5), and 6 weeks old (WF) (*n* = 5) and subjected to 16S rRNA gene sequencing analysis. Microbiota in different gut parts presented different change trends and were significantly affected by time and breastfeeding.

**FIG 3:**
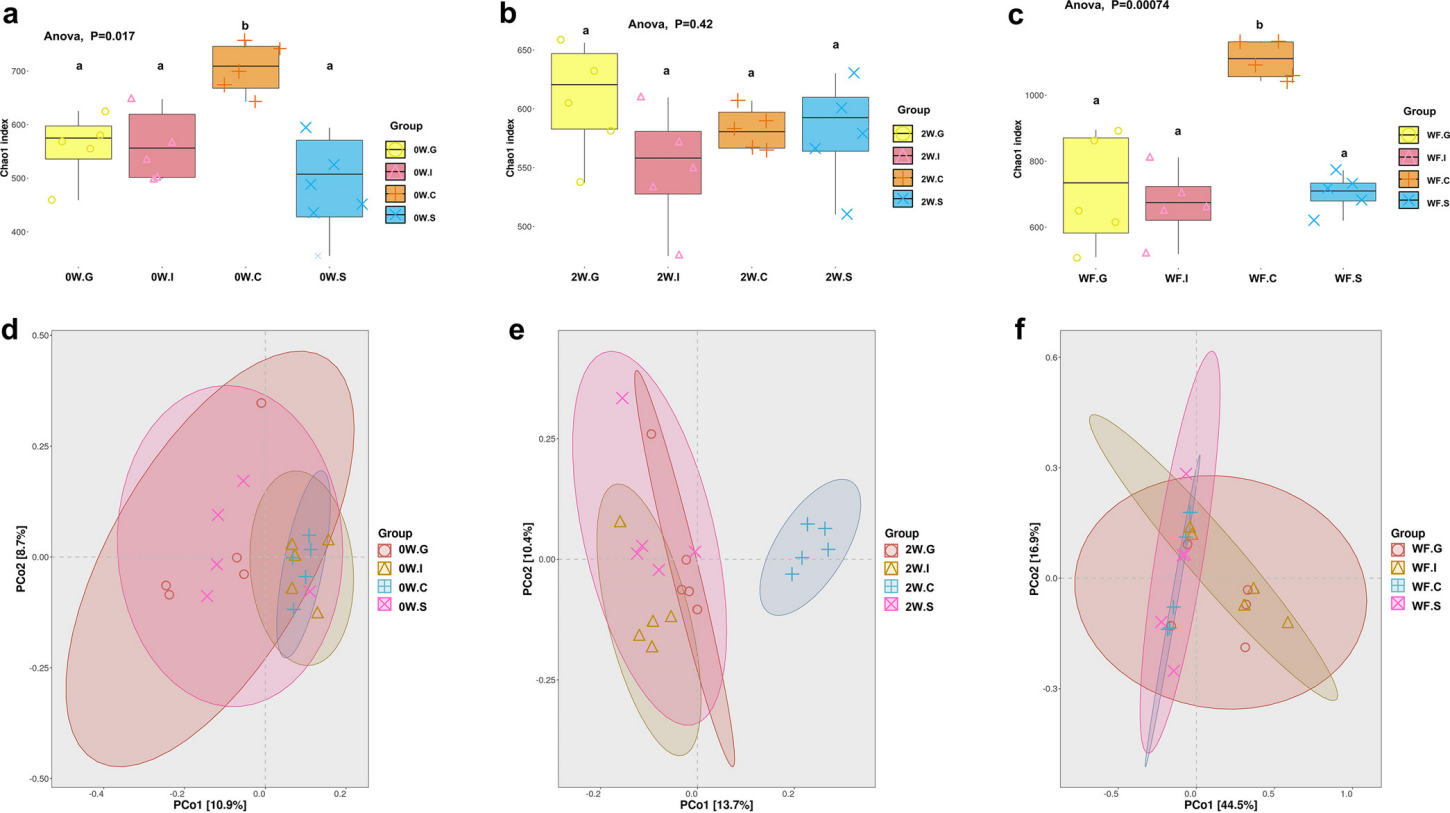
Alpha diversity and beta diversity of gastrointestinal microbiota in different periods of early life. (a) Chao1 index of gastrointestinal microbiota in the gastric mucus (G), ileum mucus (I), cecum mucus (C), and stools (S) from mice at birth (0W group). (b) Chao1 index of gastrointestinal microbiota in the G, I, C, and S from mice at 2 weeks old (2W group). (c) Chao1 index of gastrointestinal microbiota in the G, I, C, and S from mice at 6 weeks old (WF group). (d) Beta diversity was calculated among G, I, C, and S in 0W group. (e) Beta diversity was calculated among G, I, C, and S in 2W group. (f) Beta diversity was calculated among G, I, C, and S in WF group. Groups marked with the same lowercase letter were not significantly different from one another, and those marked with different letters differed significantly (*P* < 0.05) by Kruskal–Wallis tests and one-way ANOVA.

**Microbiota richness and diversity of most GI parts increased markedly in lactation period.** At birth, the cecum mucus microbiota showed the highest α-diversity indices (*P* < 0.05), followed by the ileum mucus and gastric mucus, with the stools exhibiting lower diversity (Fig. 3a, Fig. S3 and S4). At week 2, the α-diversity indices of stools microbiota presented a trend of increasing (*P* < 0.05), and those of ileum and cecum mucus showed a slight trend of decreasing (*P* < 0.05) (Fig. 3b, Fig. S3 and S4). Based on Simpson’s and Shannon indices, microbiota diversity at week 2 ranked high to low in stool, gastric mucus, cecum mucus, and ileum mucus. At week 6, microbiota diversity apparently increased in the cecum and stools, which contrasted with that in the stomach and ileum. Cecum mucus microbiota became the most abundant at week 6, followed by gastric mucus, stool, and ileal ileum mucus (Fig. 3c, Fig. S3 and S4). β-Diversity analysis presented a clustering of microbiota between the gastric mucus and the ileum mucus and a separation between the cecum mucus and the meconium at birth. Compared to week 0 and week 2 samples, the gastric mucus samples from week 6 were more dispersed. Ileum mucus, cecum mucus, and stool samples in different periods were markedly separate (Fig. 3d to f).

**FIG 4:**
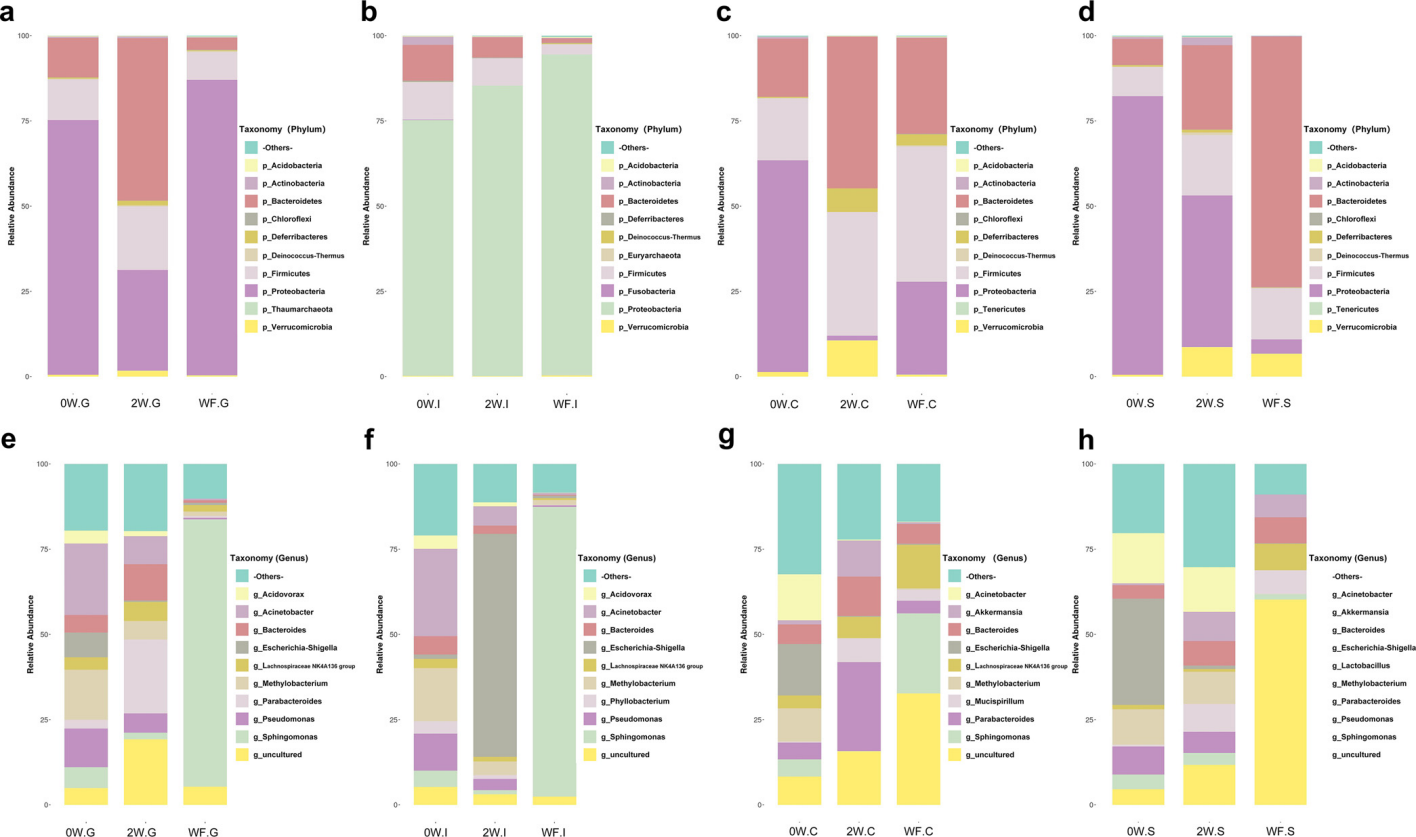
Gastrointestinal microbiota composition in different periods of early life. At phylum level, microbiota compositions of gastric mucus (a), ileum mucus (b), cecum mucus (c), and stools (d) in mice at birth (0W group), mice at 2 weeks old (2W group), and mice at 6 weeks old (WF group), respectively. At genus level, microbiota compositions of gastric mucus (e), ileum mucus (f), cecum mucus (g), and stools (h) in 0W, 2W, and WF groups, respectively.

**The microbiota structures of stools were similar to that of cecum mucus at birth, with a significant difference from those of other sites.** To explore the development of the gut microbiota in early life, relative abundances of the predominant taxa were evaluated (Fig. 4a to d). A total of 12 phyla were identified. *Firmicutes*, *Bacteroidetes*, and *Proteobacteria* were abundant (>2% of the population). The low-abundance phyla (<2% of the population) included *Verrucomicrobia*, *Deferribacteres*, *Actinobacteria*, *Deinococcus-Thermus*, *Acidobacteria*, *Chloroflexi*, *Euryarchaeota*, *Fusobacteria*, and *Tenericutes*.

At week 0, the relative abundance of *Proteobacteria* showed a slight increase from gastric mucus to gut and stools (*P* > 0.05). The relative abundances of *Firmicutes* and *Bacteroidetes* were almost equivalent in each different gut location. *Actinobacteria*, *Verrucomicrobia*, and *Deferribacteres* were less dominant in the stool and GI tract mucus microbiota. The relative abundance of *Actinobacteria* was relatively high in ileum mucus compared to that in other gut locations (*P* < 0.05). For *Verrucomicrobia*, the relative abundance in cecum mucus was significantly higher than that in other locations (*P* < 0.05).

**In lactation period, the predominant phyla were changed in cecum mucus and stools and were relatively stable in gastric and ileum mucus.** After 2 weeks of breastfeeding, the microbiota in the gut mucus and stools changed in various degrees (Fig. 4a to d). In the gastric mucus, *Proteobacteria* lost its major role, and this change was accompanied by the increase of the percentage of *Firmicutes* and *Bacteroidetes*. In the ileum mucus, the major bacterial phyla group changed from three to four dominant phyla, with the addition of *Verrucomicrobia*. The relative abundance of *Bacteroidetes* in ileum mucus decreased and was lowest in the ileum mucus compared to the other three locations (*P* < 0.05). In the cecum mucus, the relative abundance of *Proteobacteria* also showed a marked decrease (*P* < 0.05), accompanied by an increase in the percentages of *Firmicutes*, *Bacteroidetes*, and *Deferribacteres*. In stool, despite its decreased abundance, *Proteobacteria* still comprised a substantial percentage of the microbiota.

**In postweaning period, *Proteobacteria* increased as a feature in mice GI.** At week 6, the relative abundance of *Proteobacteria* conversely showed a moderate decline from gastric mucus to cecum mucus (*P* < 0.05) and stools (*P* < 0.05), respectively (Fig. 4a, c, and d). In the gastric mucus, the percentages of *Firmicutes* and *Bacteroidetes* were decreased while the percentage of *Proteobacteria* was increased after the transient drop at week 2. In the ileum mucus, the relative abundance of *Proteobacteria* continued to increase compared to that of the week 2 samples and was accompanied by a slight decrease in the relative abundance of *Firmicutes* and *Bacteroidetes* (*P* < 0.05). In the cecum mucus, the microbiota composition was approximately equal to that of each of the three major phyla (*Proteobacteria*, *Firmicutes*, and *Bacteroidetes*), plus a notable presence of *Deferribacteres*. In stools, the major phyla were *Bacteroidetes*, *Firmicutes*, *Verrucomicrobia*, and *Proteobacteria*.

**At the genus level, relative abundance of *Acinetobacter* changed noticeably in mice GI after birth.** The GI microbiota during different periods of early life were further explored at the genus level. As shown in Fig. 4e to h, microbiota composition in different parts of the gut changed over time. In the gastric mucus, the most abundant genera were *Acinetobacter* at week 0, *Parabacteroides* at week 2, and *Sphingomonas* at week 6, respectively. In the ileum mucus, the dominant genera were *Acinetobacter* at week 0, *Escherichia-Shigella* at week 2, and *Sphingomonas* at week 6, respectively. In the cecum mucus, the most abundant genera were *Escherichia-Shigella* at week 0, *Parabacteroides* at week 2, and *Sphingomonas* at week 6. In stools, the most abundant bacterial genera were *Escherichia-Shigella* at week 0, *Acinetobacter* at week 2, and *Lactobacillus* and *Bacteroides* at week 6. Compared with that in the GI microbiota in fetal mice before birth, the relative abundance of *Acinetobacter* increased noticeably in all GI parts (*P* < 0.05).

### Gastrointestinal microbiota alterations following antibiotic exposure.

Antibiotics in early life modified the indigenous microbiota of neonatal mice, and antibiotic intervention in the breastfeeding period could exert an influence on the development of gut microbiota until adulthood. Mice treated with antibiotics in the lactation period presented impressive similarities, while naive mice and mice treated with antibiotics after breastfeeding shared some patterns in relative abundance and diversity of microbiota.

**FIG 5:**
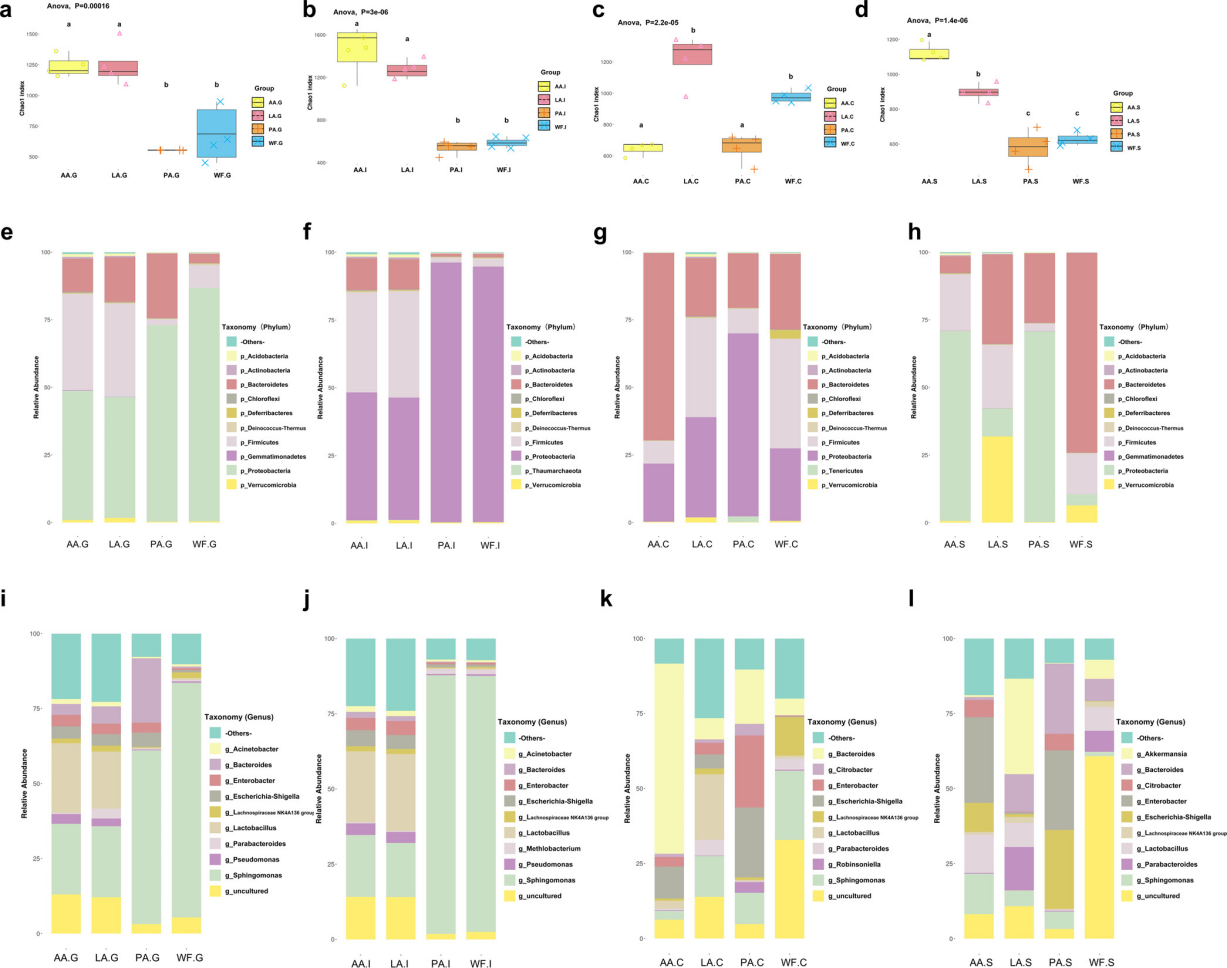
Gastrointestinal microbiota alterations following antibiotic exposure. (a) Chao1 index of gastrointestinal microbiota in the gastric mucus (G) from mice at AA, LA, PA, and WF groups. (b) Chao1 index of gastrointestinal microbiota in ileum mucus (I) from mice at AA, LA, PA, and WF groups. (c) Chao1 index of gastrointestinal microbiota in the cecum mucus (C) from mice at AA, LA, PA, and WF groups. (d) Chao1 index of gastrointestinal microbiota in the stools (S) from mice at AA, LA, PA, and WF groups. (e to h) At phylum level, microbiota compositions of gastric mucus (e), ileum mucus (f), cecum mucus (g), and stools (h) in AA, LA, PA, and WF groups. (i to l) At genus level, microbiota compositions of gastric mucus (i), ileum mucus (j), cecum mucus (k), and stools (l) in AA, LA, PA, and WF groups. Groups marked with the same lowercase letter were not significantly different from one another, and those marked with different letters differed significantly (*P* < 0.05) by Kruskal–Wallis tests and one-way ANOVA.

**Microbiota richness and diversity showed the opposite trend at different GI parts.** α-Diversity of gut microbiota was evaluated in the neonatal and antibiotic-interfered mice (Fig. 5a to d, Fig. S5). In the gastric mucus, both the AA (*n* = 4) and LA (*n* = 4) groups had higher α-diversity indices than WF (*n* = 5) and PA (*n* = 4) groups (*P* < 0.05). In the ileum mucus, the AA and LA groups showed α-diversity indices significantly higher than those of the WF and PA groups (*P* < 0.05). In the cecum mucus, the AA group showed α-diversity indices significantly lower than those of other groups. In stools, the mice microbiota diversity was highest in the AA group, and the PA group exhibited a lower diversity. Microbial community structure (β-diversity) was significantly different among the four groups (Fig. S6). The AA group and LA group were clustered, while the PA and WF groups were adjacent, with distance between AA/LA groups and PA/WF groups in the gastric mucus and ileum mucus. In the cecum mucus, samples in different groups were separated, and in stools, the PA and AA groups overlapped.

**At the phylum level, the microbiota structures also showed differences.** At week 6, the microbiota of the gastric mucus, ileum mucus, cecum mucus, and stools were examined (Fig. 5e to l). A total of 12 phyla were identified, including three high-abundance phyla, *Firmicutes*, *Bacteroidetes*, and *Proteobacteria*, and nine low-abundance phyla, *Deferribacteres*, *Verrucomicrobia*, *Tenericutes*, *Acidobacteria*, *Deinococcus*-*Thermus*, *Actinobacteria*, *Chloroflexi*, *Gemmatimonadetes*, and *Thaumarchaeota*.

In the gastric mucus (Fig. 5e), the three most abundant phyla were *Proteobacteria*, *Firmicutes*, and *Bacteroidetes*. The relative abundance of *Proteobacteria* in the PA group was significantly higher than that in the LA and AA groups (*P* < 0.05) and similar to that in the WF group (without antibiotics), accompanied by a lower relative abundance of *Firmicutes*. After antibiotic intervention, the relative abundance of *Bacteroidetes* was higher in the PA group than in other groups (*P* < 0.05), and that of *Firmicutes* was higher in the AA and LA groups.

In the ileum mucus (Fig. 5f), *Proteobacteria* comprised a great proportion in the PA groups. Similar to the LA, the relative abundance of *Proteobacteria* was decreased and accompanied by a higher relative abundance of *Firmicutes* and *Bacteroidetes* in the AA groups.

In the cecum mucus (Fig. 5g), the four most abundant phyla in the WF group were *Firmicutes*, *Bacteroidetes*, *Proteobacteria*, and *Deferribacteres*. In the PA group, the relative abundances of *Firmicutes*, *Bacteroidetes*, and *Deferribacteres* were decreased and accompanied by significantly increased abundances of *Proteobacteria* and *Tenericutes*. Relative abundances of phyla in the cecum mucus of the LA group were similar to those of the WF group, except for the disappearance of *Deferribacteres* and increase in *Verrucomicrobia*. Compared with the WF group, AA mice presented with a significant increase in *Bacteroidetes* (*P* < 0.05) and decrease in *Firmicutes* (*P* < 0.05).

In stools (Fig. 5h), the major abundant phyla in WF group (no antibiotics) were *Bacteroidetes*, *Firmicutes*, *Verrucomicrobia*, and *Proteobacteria*. In antibiotics exposure groups, *Proteobacteria* increased and became the dominant phylum in PA and AA groups. In LA group, *Proteobacteria* and *Verrucomicrobia* increased, while *Bacteroidetes* decreased.

**At the genus level, the *Lactobacillus* and *Akkermansia* genera were significantly increased after antibiotic intervention.** Microbiota were further explored at the genus level in the different groups (Fig. 5i to l). As mentioned above, the most abundant genus in WF mice in the gastric mucus was *Sphingomonas*, and following antibiotic treatment during breastfeeding, *Lactobacillus* became the dominant bacterial genus in the stomach of the mice in LA and AA groups. In the ileum mucus, *Sphingomonas* still was the most abundant bacterial genus in the PA group, but it showed a significant decrease in the LA and AA groups (*P* < 0.05). Once again, *Lactobacillus* became the dominant bacterial genus in the LA and AA groups. In stools, the dominant bacterial genus was *Bacteroides* in the WF group, but this changed to *Enterobacter*, *Escherichia-Shigella*, and *Bacteroides* in the PA group and to *Akkermansia*, *Parabacteroides*, and *Bacteroides* in the LA group. In the AA group, *Lactobacillus* became the third dominant genus in stools, right after *Enterobacterium* and *Sphingomonas*.

### Impact of antibiotics on the development of colitis.

**FIG 6:**
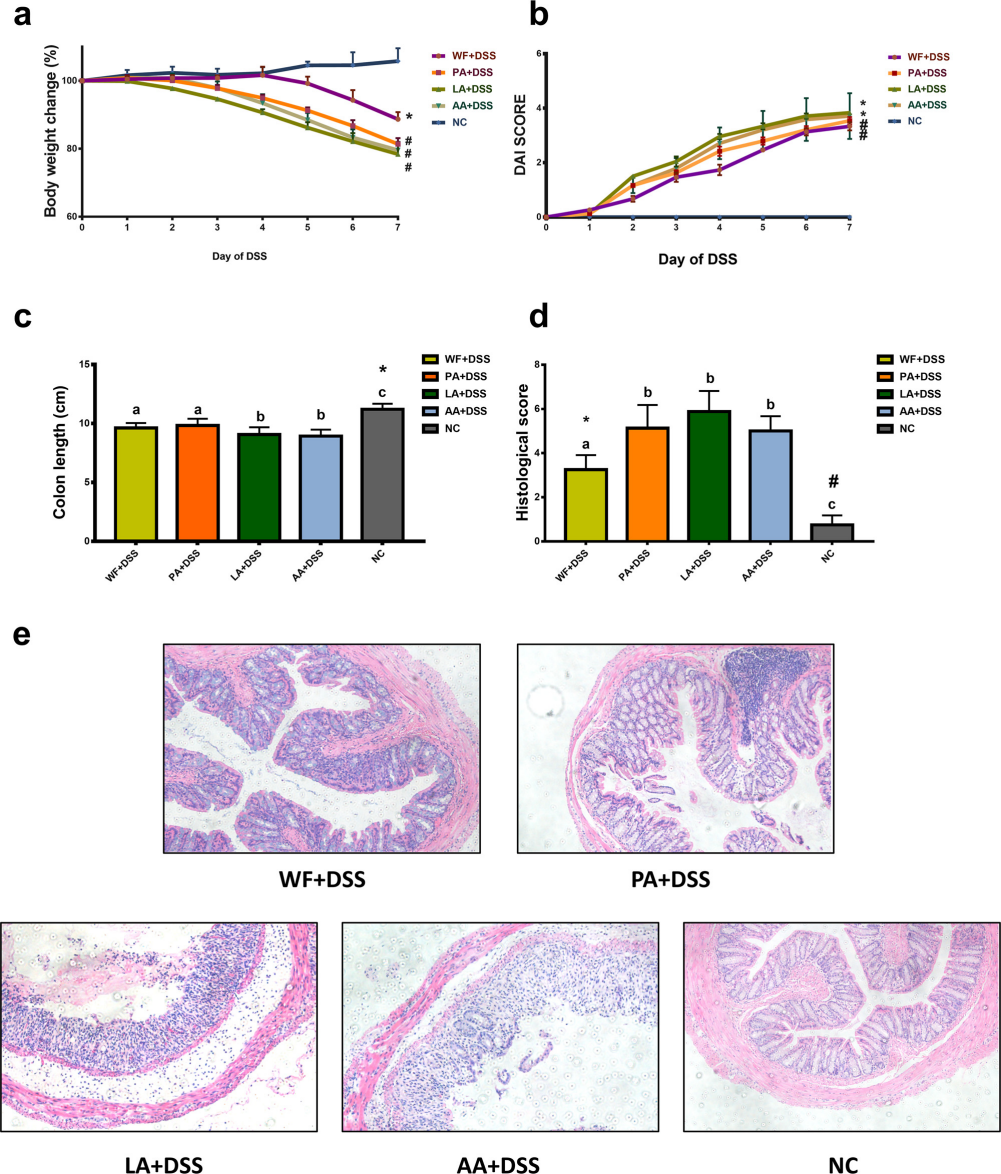
Impact of antibiotics on the development of colitis. (a) Body weight change during DSS experiment. All animals in the DSS groups lost weight, and the change of body weight was more evident in the antibiotic exposure groups than in the WF plus DSS group. *, versus NC group, *P* < 0.05; #, versus NC and WF+DSS group, *P* < 0.05. (b) DAI scores change during DSS experiment. The DAI scores for the LA plus DSS and AA plus DSS groups were higher than those of the WF plus DSS and PA plus DSS groups. *, versus NC group, *P* < 0.05; #, versus NC and WF+DSS group, *P* < 0.05. (c) The colon length of each group was measured. *, versus other groups, *P* < 0.05. (d) Histological score in NC, WF plus DSS, PA plus DSS, LA plus DSS, and AA plus DSS groups. The severity of colitis was associated with a significantly higher histological score in the antibiotic exposure groups than in the WF plus DSS group. *, WF+DSS group versus other groups, *P* < 0.05; #, NC group versus other groups, *P* < 0.05. (e) Representative images of H&E staining in the NC, WF plus DSS, PA plus DSS, LA plus DSS, and AA plus DSS groups. ×100 magnification.

The relationship of perturbed microbiota at early life and susceptibility of IBD was examined by a DSS-induced colitis mouse model. Oral administration of DSS induced acute colitis as characterized by the presence of bleeding stools, marked diarrhea, and body-weight loss in mice. These symptoms were more pronounced in the antibiotic exposure groups (PA plus DSS [*n* = 5], LA plus DSS [*n* = 5], and AA plus DSS groups [*n* = 5]) than in the WF plus DSS group (*n* = 5). All animals in the DSS groups lost weight, and the change of body weight was more evident in the antibiotic exposure groups. (Fig. 6a).

DSS-induced acute colitis resulted in a significant increase in the disease activity index (DAI) from day 2 onwards compared with that in the control group. On day 7, there was a significant difference in the DAI between the control group and the DSS groups. Among the DSS groups, the DAI scores for the LA plus DSS and AA plus DSS groups were higher than those of the WF plus DSS and PA plus DSS groups (*P* < 0.05). There was no significant difference between the DAI scores of the LA plus DSS group and AA plus DSS group and between the WF plus DSS group and PA plus DSS group (Fig. 6b).

The colon length revealed a marked shortening in the DSS-treated mice on day 7 of DSS treatment (*P* < 0.05) (Fig. 6c). The severity of colitis was associated with a significantly shorter colon length in the LA plus DSS and AA plus DSS groups compared to that in the WF plus DSS group (*P* < 0.05).

Five colon samples from each group were evaluated histologically. The antibiotic exposure groups showed a histological score significantly higher than that of the WF plus DSS group (*P* < 0.05). However, there were no differences in histological colon damage scores among the PA plus DSS, LA plus DSS, and AA plus DSS groups (Fig. 6d and e).

## DISCUSSION

Multiple studies have documented that early microbial colonization could significantly contribute to long-term health outcomes throughout a lifespan (26–28). Early life was considered to be a critical period for microbiota colonization and maturation. Therefore, the current study explored the perinatal microbiota and development of GI microbiota in each early-life period of mice, including birth, lactation, and the postweaning nutrition period.

Initial findings from this study confirmed the presence of bacteria in the GI tract of mice before birth and demonstrated that newborn mice contained microbiota similar to the maternal GI microbiota. These indicated that maternal GI microbiota might be the first seeding bacteria source in the uterus. It was assumed for many years that the fetus was maintained in a sterile state and that microbiota started colonizing the infant after birth (29, 30). The establishment of neonatal microbiota has been seen as commencing at birth, and maternal bacteria from the vagina and rectum were considered to be the seeding bacteria of the fetal microbiome (31, 32), until recent studies that employed modern sequencing technologies challenged the traditional view of human microbiome acquisition (33–35). These studies found bacterial DNA in umbilical cord blood, placenta, amniotic fluid, meconium, and fetal membranes in healthy, normal pregnancies, leading to a speculation that the seeding of the fetal microbiota may commence *in utero* long before delivery (36). Currently, the most likely hypothesis is that microbiota are translocated from the gut epithelium into the bloodstream and then delivered to the placenta. However, this hypothesis was met with skepticism because it faced the common technical issue of contamination (15, 30). In the current study, samples from different parts of the GI system were collected from fetal mice simultaneously, and the possibility of sample environment contamination was partially excluded since the parts of the GI system showed different microbiota structures instead of complete similarity. The archenteric bacteria detected were dominantly anaerobic and facultative anaerobes, which may exploit the naive conditions of the neonatal intestine. Microbiota structures in different gut parts were similar to those of corresponding gut parts of maternal mice, especially in the gastric mucus. These events may reflect a coevolutionary relationship in GI microbiota of maternal mice and fetal mice. This is congruent with previous reports that the composition and development of infant gut microbiota could be influenced by many prenatal factors, such as maternal diet, obesity, smoking status, and use of antibiotic agents during pregnancy (37). Although this study attempted to summarize the process of bacterial colonization in fetal mice by matching each region with similar microbiota structures, it still could not be concluded from existing data and should be explored in future studies.

Neonatal GI microbiota colonization is a fragile, dynamic, and stepwise process and may be affected by several maternal and neonatal factors after birth, when the first seeding bacteria in GI will be selected and matured (16). During this development period, intestinal microbiota undergo a gradual succession (7, 16, 38, 39). In the mouse model, the gut microbiota in different locations were found to undergo different developmental processes over time. At birth, α-diversity was highest in mucus of the cecum and stomach and lowest in the meconium. The composition and relative abundance of gut microbiota were largely similar to each other in the different parts of the GI tract and the meconium during this period. The low diversity of microbiota detected in the meconium was against the theory that seeding bacteria were inherited from the maternal vagina during delivery and instead supported the existence of bacteria in the prenatal environment. An interesting phenomenon was the appearance of *Acinetobacter* in mice after birth. The genus *Acinetobacter* includes a broad group of bacteria that are ubiquitous in many environments, and it is characterized by its innate and acquired antibiotic resistance (40). Notably, the bacteria also can live in antibiotic-free environments. There are several reports on detection of *Acinetobacter* in the skin, gut, and reproductive tract samples from laboratory mice (41–44). Thus, the presence of *Acinetobacter* in neonatal mice may come from the environment, not due to antibiotic use. Although samples in each mouse were routinely collected within 2 h of birth in our study, neonatal mice could become inevitably exposed to the *Acinetobacter* in the environment during this period. Natural microbiota exposed by this period is therefore thought to have shaped the GI microbiota of neonatal mice.

After 2 weeks of breastfeeding, microbiota in the gut mucus and stools changed to different degrees. In line with previous studies (16, 20, 45), microbiota maturation was accelerated during breastfeeding. With the degradation of complex diets, microbiota diversity increased and the composition changed to be bacterial genera specialized. In gastric and cecum mucus, *Proteobacteria* decreased and *Bacteroidetes* increased during breastfeeding, while in ileum mucus, the microbiota structure changed from three dominant bacterial phyla groups to four. The microbiota diversity presented with alterations toward separate directions in different GI parts, indicting the dynamic process of mucus microbiota maturation. All these results suggested the importance of the lactation period, and this period paralleled the diet of breastmilk, which is an important source of introduced microbes. It has previously been shown that the composition of the gut microbiota underwent changes from the mucosal to the luminal/fecal side (46). This was also supported in the present study by the different microbiota diversity changes in stool and gut mucus. These findings suggest that exploration of the mucus microbiota should be required, since the research restricted to fecal samples cannot fully represent the bacterial variations in the gut.

At week 6, the microbiota of mice gradually stabilized toward an adult-like community, characterized by higher diversity in the cecum mucus and lower diversity in the gastric mucus, ileum mucus, and stools. When breastfeeding is ceased, most nutrients from food are digested and absorbed in the small intestine. Hence, GI microbiota changes toward bacterial genera specialized in degrading complex dietary carbohydrates (16). In accord with our observations, the cecum microbiota diversity increased. However, it was surprising that microbiota diversity in stools decreased by 6 weeks of age, despite the idea that more bacteria should be present with the consumption of solid food. The different trends between cecum mucus and stools hinted that gut microbiota need to be defined more accurately. In addition, the exact composition of the mucosal microbiota remains poorly studied in contrast to the fecal gut microbiota, and further research is required to address this question.

After exploring the three distinct phases during early life, further investigations were conducted to determine the critical period of GI microbiota development in mice by using antibiotic-interfered and DSS-induced colitis mice model. Our research revealed that lactation-period antibiotics-exposed mice presented marked similarity in the gastric and ileum mucus microbiota, while no lactation-period antibiotics exposure groups shared a mild similar pattern in microbiota in these gut locations, even with antibiotics after breastfeeding. Furthermore, whether exposed to antibiotics after lactation period or during the whole process, mice all presented similar microbiota in cecum mucus. Given that, the lactation period seemed to be more important for the development of gastric and ileum mucus microbiota, as the postweaning nutrition period is for cecum mucus. A possible explanation for this fact might be that solid food produces more metabolites in the cecum than breast milk, and it is in accordance with the evidence that high-fat and high-fiber food could affect gut microbiota in stools (25). Antibiotic interference in the lactation period resulted in increased abundance and diversity of microbiota in the stomach and ileum mucus. Previous studies on antibiotic-exposed microbiota have found that the α-diversity was reduced in stool samples after the intervention (47, 48), while the host microbiota in different GI parts could have different proliferative capacities after antibiotic treatment (49). Moreover, it can be varied with different antibiotic types (47, 49). Long-term use of antibiotics can cause the decrease of dominant flora and overgrowth of foreign flora or nonpathovar flora, which may result in a further increase in α-diversity (50, 51). The complex relationship among microbiota diversity, GI site, and duration of antibiotics uses as well as antibiotic type should be taken into further studies. By evaluating the severity of colitis with DAI score and histological evaluation, the antibiotics-exposed group in the lactation period exhibited increased severity of colitis. The importance of the lactation period in microbiota development and relative disease susceptibility was verified. Similar to our research, Munyaka et al. (52) investigated the role of antepartum antibiotics and presented enhanced DSS-colitis severity and perturbed offspring microbiota in their study.

There are some limitations in the present study. One limitation of our study was the lack of an operating theater negative control (swab exposed to operating theater air), which could offset the data but does not affect conclusions. Second, after confirming the presence of bacteria in the GI tract of mice before birth, the origin of the fetal mice GI microbiota could not be traced further due to limitations in the microbiota analysis methods. Third, only normal mice were used to apply to real life with a more diverse microbiome. Fecal microbiota transplantation in germfree or gnotobiotic mice may provide additional evidence for further studies. In addition, DSS-induced colitis is a chemically induced inflammation, which can only partially recapitulate human IBD.

In conclusion, this study investigated resident bacteria in the whole GI tract to explore gut microbiota development in early life. Early-life antibiotic exposure exacerbated alterations in gut microbiota and murine DSS-induced colitis. Furthermore, the presence of bacteria in GI tract of mice before birth and the importance of the lactation period in GI microbiota development were confirmed. The microbial populations and dynamics revealed in the study deserve further scrutiny for their role in host-microbial communication, host development, and health.

## MATERIALS AND METHODS

### Animals.

All animal experiments and procedures were approved by Animal Care Committee of Central South University, Central South University, Changsha, Hunan, China.

**FIG 7:**
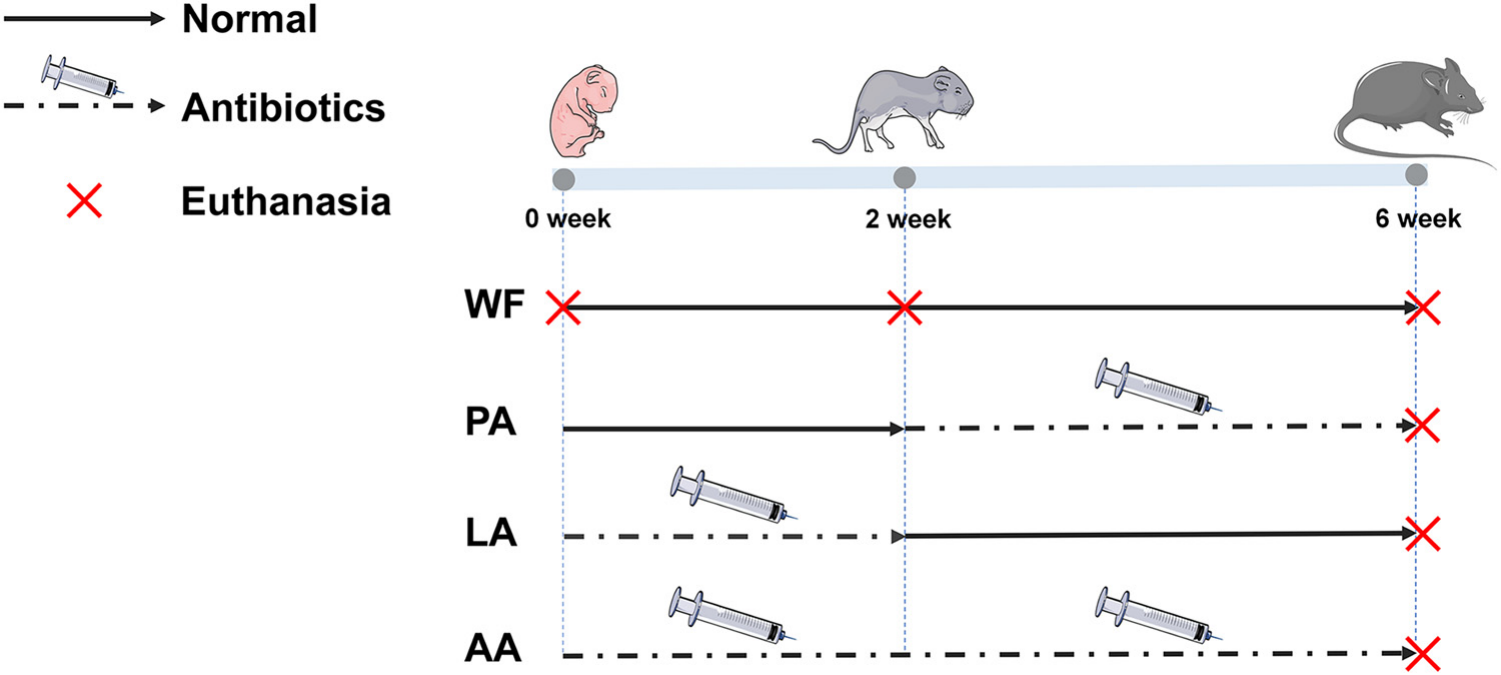
Experimental plan and treatment groups. WF group pups were raised with regular breastfeeding for 2 weeks and then were transferred to a normal chow diet after weaning until the age of 6 weeks. PA group: pups were raised with normal breastfeeding for 2 weeks. The mother mice were then administered with antibiotics by gavage from day 15 to day 21, and breastfeeding continued until pups weaned at 3 weeks old. Pups were then treated with antibiotics by gavage from week 4 until week 6. LA group: mother mice were treated with antibiotics by gavage for 2 weeks after giving birth and kept breastfeeding pups. After cessation of antibiotic treatment, breastfeeding was continued until weaning at day 21. Pups were then fed with a regular chow diet until the age of 6 weeks. AA group: mother mice were administered with antibiotics by gavage for 3 weeks from giving birth until weaning and pups were breastfed during this time. Pups were then treated with antibiotics by gavage from week 4 until week 6.

Experimental plan and treatment groups are shown in Fig. 7. Eighteen pregnant C57BL/6 mice (1 week before the expected date) were purchased from Hunan Slack Jingda Laboratory Animal Technology Company and housed individually with unlimited access to food in a 12-h light/dark cycle. Pups of each litter were raised in the same cage as the mother, and the following groups were established.

WF group (no antibiotic intervention, *n* = 9 cages): pups were raised with regular breastfeeding for 2 weeks and then were transferred to a normal chow diet after weaning until the age of 6 weeks.

PA group (postweaning antibiotic intervention, *n* = 3 cages): pups were raised with normal breastfeeding for 2 weeks. The mother mice were then administered with antibiotics (cefazolin 160 mg/kg/day, ampicillin 340 mg/kg/day) by gavage from day 15 to day 21, and breastfeeding continued until pups weaned at 3 weeks old. Pups were then treated with antibiotics by gavage from week 4 until week 6.

LA group (lactation antibiotic intervention, *n* = 3 cages): mother mice were treated with antibiotics (cefazolin 160 mg/kg/day, ampicillin 340 mg/kg/day) by gavage for 2 weeks after giving birth and kept breastfeeding pups. After cessation of antibiotic treatment, breastfeeding was continued until weaning at day 21. Pups were then fed with a regular chow diet until the age of 6 weeks.

AA group (postnatal antibiotic intervention, *n* = 3 cages): mother mice were administered with antibiotics (cefazolin 160 mg/kg/day, ampicillin 340 mg/kg/day) by gavage for 3 weeks from giving birth until weaning, and pups were breastfed during this time. Pups were then treated with antibiotics by gavage from week 4 until week 6.

### Induction of DSS-induced colitis.

A DSS-induced colitis model was used to assess the disruptive effect of antibiotics on disease severity. DSS (molecular weight 40 kDa; MP Biomedicals, Soho, OH, USA) was added to drinking water at a final concentration of 2.5% (wt/vol) and given to WF, PA, LA, and AA groups of mice for 6 consecutive days, and then each group was changed to regular drinking water for 1 day. A control group from the WF group was given regular drinking water for 7 days.

### Sample collection and assessment of clinical disease.

Mice were anesthetized and sacrificed at individual time points upon the termination of the experiment. Five pregnant mice were sacrificed on the expected date of confinement before giving birth. Mice in the WF group were sacrificed at birth (0W; within 2 h after birth), 2 weeks old (2W), 6 weeks old (WF), 7 weeks old (DSS control group), and after DSS intervention, respectively. Mice in the PA, LA, and AA groups were sacrificed at 6 weeks old and after DSS intervention, respectively. Since the number of birth mice per cage varied, the experimental groups included more than five mice from each litter. The experiment was repeated three times, with more than three mice per experimental group.

The stomach of each mouse was excised and gently washed with sterile saline after gastric contents were removed by cotton swab. A gastric mucus sample was collected by gently scraping and was frozen in liquid nitrogen. Cecum mucus, ileum mucus, and feces were collected in the same way. Fetal mice samples comprised 5 to 9 tissues from a single litter. Colonic tissue was collected at the end of the DSS intervention and was placed into histological cassettes, fixed in 10% formalin, embedded in paraffin, and processed.

Mice were monitored daily during DSS intervention to assess disease activity. The disease activity index (DAI) comprised the score for the percentage of body weight lost in combination with scores for stool consistency and blood in feces, calculated as

DAI=(weight score + stool consistency score +  blood in feces score)/3

The scoring system for weight was as follows: 0, no loss; 1, 5 to 10% weight loss; 2, 10 to 15%; 3, 15 to 20%; and 4, >20%. Stools were scored as 0, normal, 2, loose stool, and 4, diarrhea. The scoring system for feces bleeding was 0, no blood, 2, presence of blood, and 4, gross blood. DAI scoring was performed from day 0 to day 6 over the period of DSS treatment. The presence of blood in the stool was assessed using a Hemoccult II test (Beckman coulter, Oakville, ON, Canada).

### Histopathology.

Colonic tissue samples were embedded in paraffin, and hematoxylin and eosin (H&E) staining was performed on 5-μm colon sections. Colonic damage was assessed based on a published scoring system that considered degree of inflammation (0, none; 1, mild inflammation; 2, severe inflammation), lesion depth (0, none; 1, submucosal; 2, muscular layer; 3, serosal layer), range of lesion (0, none; 1, 1 to 25%; 2, 26 to 50%; 3, 51 to 75%; 4, 76 to 100%), and crypt damage (0, none; 1, one-third crypt destruction in base region; 2, two-thirds crypt destruction in base region; 3, crypt destruction but epithelium remains intact; 4, crypt and epithelium destruction). Macroscopic and histological damages were recorded and scored for each mouse by two different investigators who were blinded to the treatment conditions. Total histological scores and individual features were averaged per group, and statistical significance was calculated by the Mann–Whitney U test.

### DNA extraction and PCR amplification.

Total genomic DNA from samples was extracted according to the descriptions. To ensure good detection and avoid contamination, one mock bacterial community standard (ZymoBIOMICS, no. D6300, Zymo Research, Murphy Ave, Irvine, CA, USA) and a blank reagent were used for quality control in each batch. DNA concentration and quality were checked using a NanoDrop spectrophotometer. DNA was diluted to 10 ng/μL using sterile ultrapure water and stored at −80°C for downstream use. The V4 region of the 16S rRNA gene was amplified with primers 515F (5′-GTGYCAGCMGCCGCGGTAA-3′) and 806R (5′-GGACTACHVGGGTWTCTAAT-3′) (53). 16S rRNA genes were amplified using the specific primer with a 12-nucleotide (nt) unique barcode. The PCR mixture (50 μL) contained 2× PCR buffer, 1.5 mM MgCl_2_, 0.4 μM each deoxynucleoside triphosphate (dNTP), 1.0 μM each primer, 0.5 U of KOD-Plus-Neo (TOYOBO), and 10 ng template DNA. The PCR amplification program consisted of an initial denaturation at 94°C for 1 min, 30 cycles of denaturation at 94°C for 20 s, annealing at 55°C for 30 s, and elongation at 72°C for 30 s, followed by a final extension at 72°C for 5 min. Three replicates of PCRs for each sample were combined for the analysis of products. PCR products mixed with 1/6 volume of 6× loading buffer were loaded on 2% agarose gels for detection. Samples with a bright major band between 200 to 400 bp were selected for further experiments. The electrophoresis bands were purified using an OMEGA gel extraction kit (Omega Bio-Tek, USA). Purified PCR products were quantified with a Qubit 2.0 (ThermoFisher) or GE NanoVue system (GE Healthcare) and mixed as required. PCR products from different samples were pooled in equal molar amounts.

### Library preparation and sequencing data analysis.

An Illumina TruSeq DNA PCR-free sample prep kit (FC-121-3001/3003) was used for library construction and index codes were added. The resulting library was subjected to paired-end sequencing (2 by 250 bp) with an Illumina Hiseq rapid SBS kit v2 (FC-402-4023 500 Cycle). Sequences were analyzed according to the USEARCH tool (http://drive5.com/uparse/) and QIIME pipeline (54). Paired-end reads from the original DNA fragments were merged using FLASH (55), and then sequences were assigned to each sample according to the unique barcode.

### OTU clustering and taxonomy assignment.

Based on USEARCH (http://drive5.com/uparse/) software, sequences were clustered into operational taxonomic units (OTUs) at 97% identity threshold using UPARSE algorithms (56). Representative sequences for each OTU were selected based on the most abundant sequence for each OTU. Taxonomy was assigned using UCLUST (57) and the SILVA database (58) (SILVA SSU 132 update release).

### Statistical methods.

To remove the influence of sequencing depth on community diversity, the OTU table was rarefied so all samples held the same number of sequences. All data analyses were performed using R, Python (https://www.python.org/), or SPSS 24.0. Alpha diversity (α-diversity) was calculated in Vegan. Unweighted Unifrac distances were calculated in GUniFrac. Principal coordinate analysis (PCoA) was performed using the Ape package (59). The between-group variance was analyzed with *t* tests, permutational multivariate analysis of variance (PERMANOVA), Kruskal–Wallis rank sum test, one-way analysis of variance (ANOVA) (normal distribution), or Mann–Whitney U test (abnormal distribution). Data were presented as means ± standard error of the mean (SEM). *P* < 0.05 was considered statistically significant.

### Data availability.

The raw sequencing data of this study have been deposited in the National Center of Biotechnology Information (NCBI) Sequence Read Archive (SRA) database under the BioProject accession number PRJNA770920. All data generated within this study are available from the corresponding author on request.
