# Supplementary material for: Development of Early-Life Gastrointestinal Microbiota in the Presence of Antibiotics Alters the Severity of Acute DSS-Induced Colitis in Mice
Source: Microbiol Spectr. 2022 Apr 19;10(3):e02692-21. doi: 10.1128/spectrum.02692-21 (PMC9241904; doi:10.1128/spectrum.02692-21)
Supplement: SUPPLEMENTAL FILE 1 — Supplemental material. Download spectrum.02692-21-s001.pdf, PDF file, 3.2 MB [file spectrum.02692-21-s001.pdf]

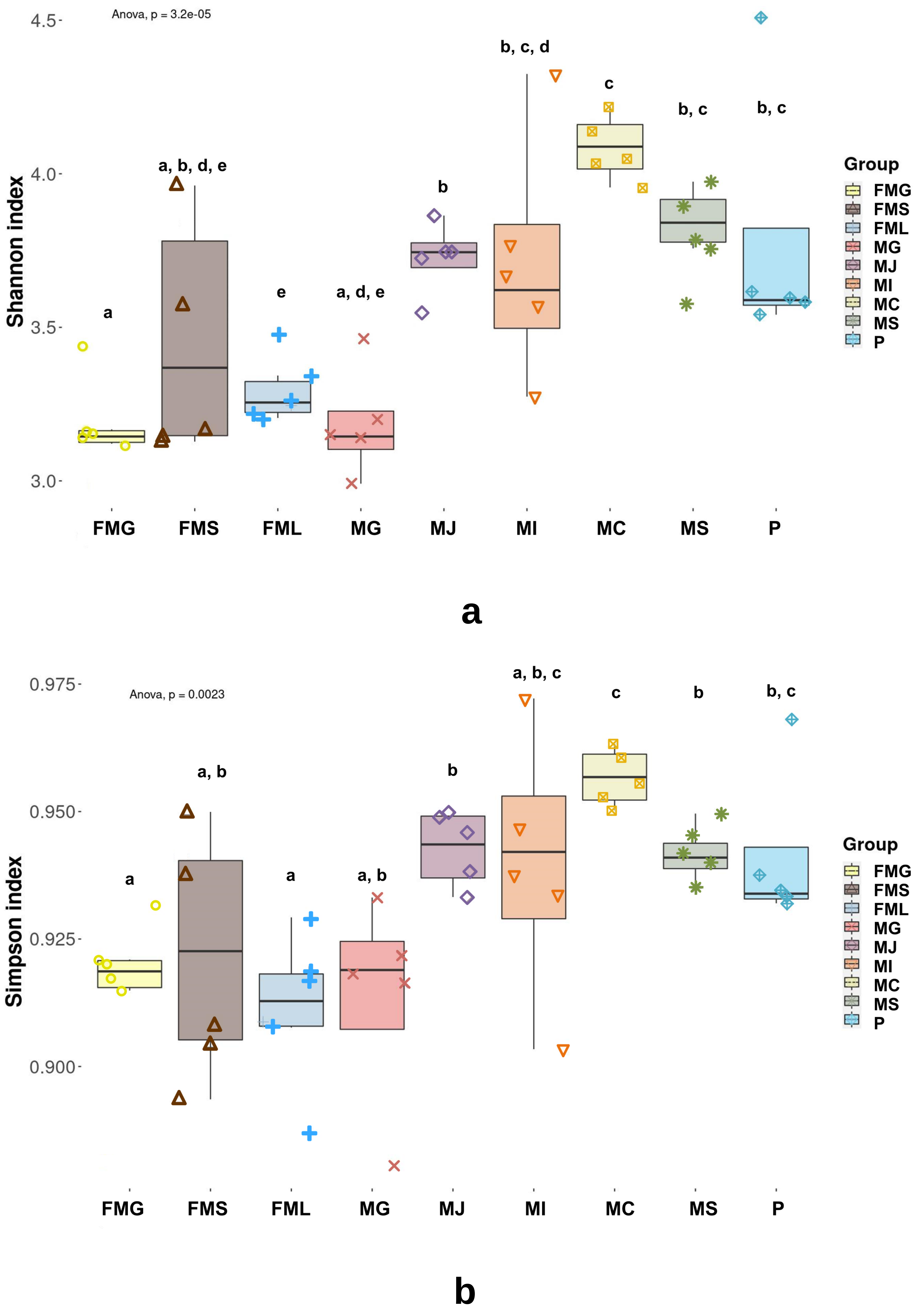

Fig.S1. Shannon (a) and Simpson (b) indices of maternal and fetal mice samples (MG, maternal gastric mucus group; MJ, maternal jejunum mucus group; MI, maternal ileum mucus group; MC, maternal cecum mucus group; MS, maternal stool group; P, placenta group; FMG, fetal mice gastric group; FMS, fetal mice small intestine group; FML, fetal mice large intestine group). Data are shown as the mean ± SEM. Groups marked with the same lower-case letter were not significantly different from one another, and those marked with different letters differed significantly ($P < 0.05$) by Kruskal–Wallis tests and one-way ANOVA.

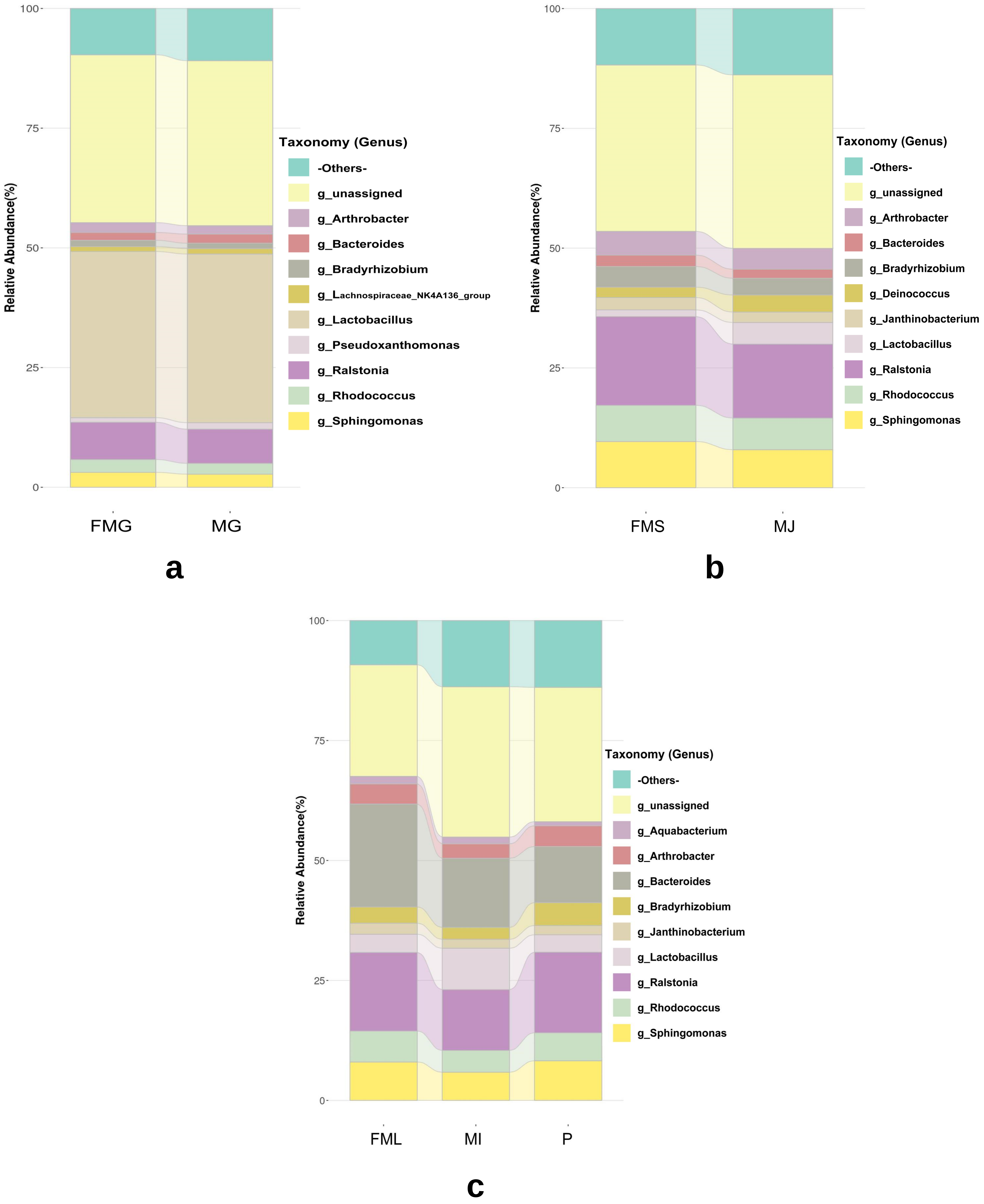

Fig.S2. Microbiota composition of maternal and fetal gastrointestinal bacteria in mice.(a) The relative abundance of bacterial in fetal mice gastric (FMG) and maternal gastric mucus (MG) at the genus level. (b) The relative abundance of bacterial in fetal mice small intestine (FMS) and maternal jejunum mucus (MJ) at the genus level. (c) The relative abundance of bacterial in fetal mice large intestine (FML), maternal ileum mucus (MI), and placenta (P) at the genus level.

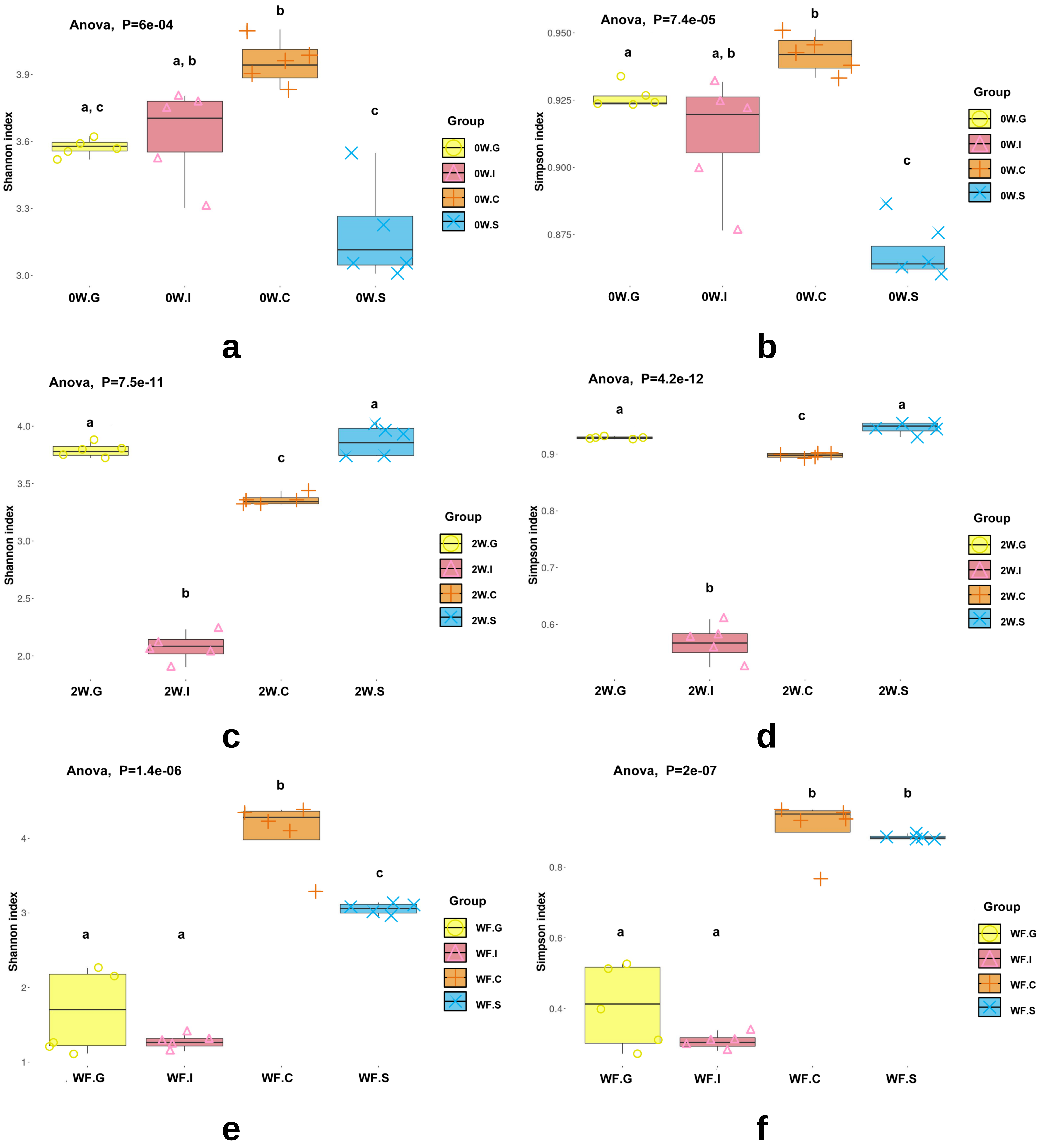

Fig.S3. Shannon (a) and Simpson (b) indices of gastrointestinal microbiota in the gastric mucus (G), ileum mucus (I), cecum mucus (C), and stools (S) from mice at birth (0W group). Shannon index (c) and Simpson index (d) of gastrointestinal microbiota in the G, I, C, and S from mice at 2 weeks old (2W group). Shannon index (e) and Simpson index (f) of gastrointestinal microbiota in the G, I, C, and S from mice at 6 weeks old (WF group). Data are shown as the mean ± SEM. Groups marked with the same lower-case letter were not significantly different from one another, and those marked with different letters differed significantly ($P < 0.05$) by Kruskal–Wallis tests and one-way ANOVA.

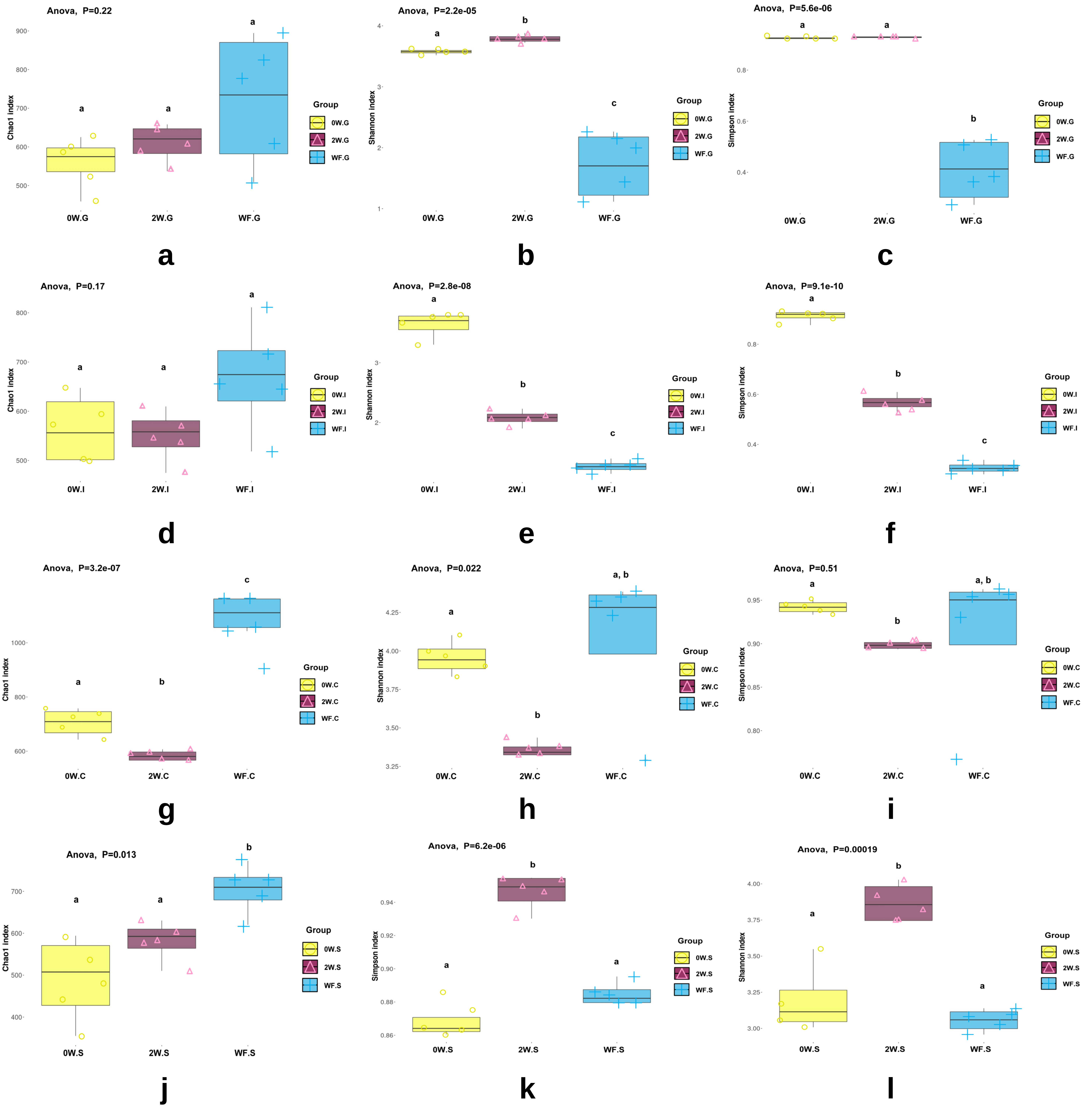

Fig.S4. Chao1 index, Shannon index, and Simpson index of microbiota in the gastric mucus (a, b, c),ileum mucus (d, e, f), cecum mucus (g, h, i), and stools (j, k, l) from mice at birth (0W group), 2 weeks old (2W group), and 6 weeks old (WF group), respectively. Data are shown as the mean ± SEM. Groups marked with the same lower-case letter were not significantly different from one another, and those marked with different letters differed significantly ($P < 0.05$) by Kruskal–Wallis tests and one-way ANOVA.

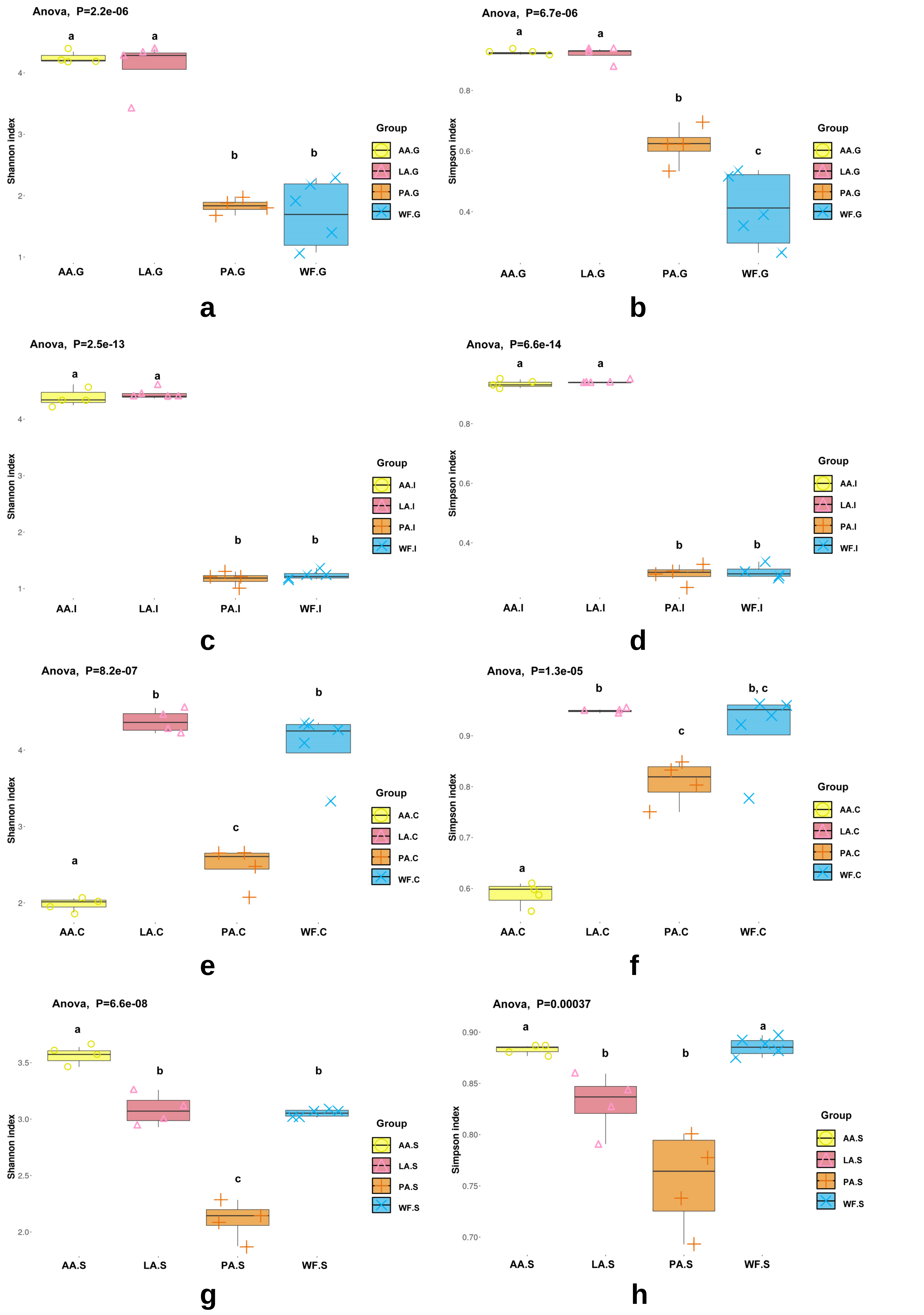

Fig.S5. Shannon index and Simpson index of microbiota in the gastric mucus (a,b), ileum mucus (c, d), cecum mucus (e, f), and stools (g, h) from AA group, LA group, PA group and WF group mice, respectively. Data are shown as the mean ± SEM. Groups marked with the same lower-case letter were not significantly different from one another, and those marked with different letters differed significantly ($P < 0.05$) by Kruskal–Wallis tests and one-way ANOVA.
